# Frequent Alteration of Annexin A9 and A10 in HPV-Negative Head and Neck Squamous Cell Carcinomas: Correlation with the Histopathological Differentiation Grade

**DOI:** 10.3390/jcm8020229

**Published:** 2019-02-10

**Authors:** Cecilia Salom, Saúl Álvarez-Teijeiro, M. Pilar Fernández, Reginald O. Morgan, Eva Allonca, Aitana Vallina, Corina Lorz, Lucas de Villalaín, M. Soledad Fernández-García, Juan P. Rodrigo, Juana M. García-Pedrero

**Affiliations:** 1 Department of Otolaryngology, Hospital Universitario Central de Asturias and Instituto de Investigación Sanitaria del Principado de Asturias, Instituto Universitario de Oncología del Principado de Asturias, University of Oviedo, Avda. Roma, 33011 Oviedo, Spain; mariaceciliasalom@gmail.com (C.S.); saul.teijeiro@gmail.com (S.Á.-T.); ynkc1@hotmail.com (E.A.); 2 CIBERONC, Av. Monforte de Lemos 3-5, 28029 Madrid, Spain; clorz@ciemat.es; 3 Department of Biochemistry and Molecular Biology and Institute of Biotechnology of Asturias, University of Oviedo, Julian Clavería, 33006 Oviedo, Spain; pfernandez@uniovi.es (M.P.F.); morganreginald@uniovi.es (R.O.M.); 4 Department of Pathology, Hospital Universitario Central de Asturias and Instituto Universitario de Oncología del Principado de Asturias, University of Oviedo, Avda. Roma, 33011 Oviedo, Spain; alaicla@hotmail.es (A.V.); solefghdr@hotmail.com (M.S.F.-G.); 5 Molecular Oncology Unit, CIEMAT (ed 70A), Av. Complutense 40, 28040 Madrid, Spain; 6 Department of Oral Surgery, Hospital Universitario Central de Asturias and Instituto de Investigación Sanitaria del Principado de Asturias, Instituto Universitario de Oncología del Principado de Asturias, University of Oviedo, Avda. Roma, 33011 Oviedo, Spain; lvillalain@hotmail.com

**Keywords:** annexin A9, annexin A10, head and neck squamous cell carcinoma, differentiation grade, immunohistochemistry

## Abstract

The annexin protein superfamily has been implicated in multiple physiological and pathological processes, including carcinogenesis. Altered expression of various annexins has frequently been observed and linked to the development and progression of various human malignancies. However, information is lacking on the expression and clinical significance of annexin A9 (ANXA9) and A10 (ANXA10) in head and neck squamous cell carcinomas (HNSCC). ANXA9 and ANXA10 expression was evaluated in a large cohort of 372 surgically treated HPV-negative HNSCC patients and correlated with the clinicopathologic parameters and disease outcomes. Down-regulation of ANXA9 expression was found in 42% of HNSCC tissue samples, compared to normal epithelia. ANXA9 expression in tumors was significantly associated with oropharyngeal location and histological differentiation grade (*p* < 0.001). In marked contrast, ANXA10 expression was absent in normal epithelium, but variably detected in the cytoplasm of cancer cells. Positive ANXA10 expression was found in 64% of tumors, and was significantly associated with differentiation grade (*p* < 0.001), being also more frequent in oropharyngeal tumors (*p* = 0.019). These results reveal that the expression of both ANXA9 and ANXA10 is frequently altered in HNSCC and associated to the tumor differentiation grade, suggesting that they could be implicated in the pathogenesis of these cancers.

## 1. Introduction

Twelve annexins comprise a ubiquitous, multigene family in vertebrates with properties that enable binding interactions with calcium and cell membrane components, including anionic phospholipids, cytoskeletal proteins and extracellular matrix glycoproteins. Annexin-knockdown or annexin-knockout models have provided limited insight into the biological functions of different annexin proteins [1] and there are only indirect links based on statistical association with genetic diseases. They have been implicated in a variety of biological processes, including membrane organization, vesicle trafficking, calcium metabolism, cell adhesion, subcellular transport, growth and differentiation, and wound healing [2,3], many of which are relevant to cancer progression.

Annexins are characterized structurally by a conserved C-terminal core that consists of a tetrad of homologous annexin (ANX) domains, each 68–69 amino acids long, harboring ligands that can coordinate calcium ions in conjunction with membrane phospholipids, or bind to other proteins and carbohydrate-containing biomolecules. The binding properties and targets of each annexin are distinct, exemplified by the apparent calcium-independence of annexins A9 and A10 [4]. The N-terminal region of each annexin is unique, with a variable length and amino acid sequence that contributes to annexin conformation, protein interactions and non-overlapping functional specificity in the biological activity of different annexins [5,6].

More than 4000 annexins have been reported in different species, widely distributed among eukaryotes and prevalent in different forms of prokaryotes and unicellular eukaryotes [1,4]. The twelve annexins common to vertebrates are referred to as annexins A1–A13 (ANXA1–ANXA13) with ANXA12 remaining unassigned. There are 13 human annexin genes, including a unique duplication of ANXA8, ranging in size from 15 kb (ANXA9) to 96 kb (ANXA10) and spread throughout the genome on chromosomes 1, 2, 4, 5, 8, 9, 10 and 15 [1,7]. 

The expression pattern and tissue distribution of annexins vary widely. While annexins A1, A2, A4, A5, A6, A7 and A11 are ubiquitously expressed, others exhibit very restrictive expression such as ANXA3 in neutrophils, ANXA8 in placenta and skin, ANXA9 in the tongue, ANXA10 in the stomach and ANXA13 in the small intestine [7]. The promoter regulation of annexin A9 has been partially characterized [8], but distal DNA elements, regulatory RNAs and epigenetic changes are under current study in high-throughput experiments, so the molecular basis of its expression remains incomplete.

The term annexinopathy has been used to define those human diseases in which abnormal levels and pleiotropic effects of annexins contribute to the pathogenesis [9,10]. Although direct involvement of these proteins in the etiology of any genetic disease has not been demonstrated, they have been implicated in various pathologies such as diabetes, cardiovascular and autoimmune diseases, infection and cancer [10,11]. Mounting evidence shows that several annexins are frequently altered in cancers, suggesting a possible role in the process of tumorigenesis. Some annexins have been found overexpressed in specific types of tumors, while others consistently show loss of expression [9,10,11]. Emerging mechanistic studies are helping to relate annexin expression changes to tumor cell function, particularly tumor growth, invasion and metastasis, angiogenesis and drug resistance. The expression of individual annexins is associated with particular cancer types hence annexins could also be useful biomarkers in the clinic [10,11]. More precise localization of these proteins in different tissues could deepen our understanding of their pathophysiological functions, which continues to be a key area of investigation.

The overall goal of this study was to investigate the expression pattern and clinical significance of ANXA9 and ANXA10, specifically in head and neck squamous cell carcinomas (HNSCC). ANXA9 shows generally restricted tissue expression but is known to exhibit altered expression in breast cancer [12], colorectal cancer [13] and cutaneous melanoma [14]. It was also shown to be overexpressed in differentiating keratinocytes in pemphigus [15] and binds to other cytoskeletal proteins [16]. Several studies have been published to date on the expression ANXA10 in gastrointestinal cancers, and its overexpression in oral cancer is correlated with cell proliferation [17]. We focused our study on the expression and clinical significance of ANXA9 and ANXA10 specifically in HNSCC using immunohistochemistry techniques in a large homogeneous cohort of 372 surgically treated, HPV-negative, HNSCC patients.

## 2. Materials and Method

### 2.1. Patients and Tissue Specimens

Surgical tissue specimens from 372 patients with HPV-negative HNSCC who underwent resection of their tumors at the Hospital Universitario Central de Asturias between 1990 and 2009 were retrospectively collected, in accordance to approved institutional review board guidelines. All experimental protocols were approved by the Institutional Ethics Committee of the Hospital Universitario Central de Asturias and by the Regional CEIC (Comité Ético de Investigación Clínica) from Principado de Asturias (approval number: 81/2013 for the project PI13/00259). Informed consent was obtained from all patients. Representative tissue sections were obtained from archival, paraffin-embedded blocks and the histological diagnosis was confirmed by an experienced pathologist (M.S.F.-G). 

All patients had a single primary tumor, microscopically clear surgical margins and received no treatment prior to surgery. Only fourteen patients were women, and the mean age was 58.6 years (range 30 to 86 years). All but twelve patients were habitual tobacco smokers, 198 moderate (1–50 pack-year) and 153 heavy (>50 pack-year), and 335 were alcohol drinkers. The stage of the tumors was determined according to the TNM system of the International Union Against Cancer (7th Edition). Two hundred and thirty (62%) of 372 patients received postoperative radiotherapy. Patients were followed-up for a minimum of 36 months. The mean follow-up for the whole series was 34.6 months (median, 21.5 months); for the patients without recurrence, 71 months (median, 67 months); and for the patients dead by the tumor, 18 months (median, 13.5 months). Recurrence was defined as relapse of the tumor in the five first years after treatment at any site: local recurrence, nodal metastasis, or distant metastasis. Information on HPV status was available for all the patients. HPV status was analyzed using p16-immunohistochemistry, high-risk HPV DNA detection by in situ hybridization and genotyping by GP5+/6+-PCR, as previously reported [18,19]. The characteristics of the studied cases are shown in Table 1.

### 2.2. Tissue Microarray (TMA) Construction

Three morphologically representative areas were selected from each individual tumor paraffin block. Subsequently, three 1 mm cylinders were taken to construct TMA blocks, as described previously [20,21], containing a total of 372 HNSCC (134 tonsillar, 107 base of tongue, 64 hypopharyngeal and 67 laryngeal carcinomas). In addition, each TMA included three cores of normal epithelium as an internal negative control. The normal epithelium was obtained from adult male, non-smokers and non-drinkers, patients that were operated from tonsillectomy due to chronic tonsillitis, and patients operated from benign vocal cord lesions (e.g., polyps, cysts).

### 2.3. Immunohistochemical Study

The formalin-fixed, paraffin-embedded tissue samples were cut into 3-μm sections and dried on Flex IHC microscope slides (Dako, Glostrup, Denmark). The sections were deparaffinized with standard xylene and hydrated through graded alcohols into water. Antigen retrieval was performed with proteinase K and the samples were placed for 15 min in hydrogen peroxide at 3%. Staining was done at room temperature on an automatic staining workstation (Dako Autostainer Plus) using the following primary antibodies (developed by Dr. MP Fernández, Department of Biochemistry, University of Oviedo [4]) and conditions: Anti-ANXA9 at a concentration of 1:100 for 30 min and anti-ANXA10 at a concentration of 1:100 for 45 min. Immunodetection was carried out with the Dako EnVision Flex + Visualization System (Dako Autostainer), using diaminobenzidine as a chromogen. Counterstaining with hematoxylin for 7 min was the final step.

After staining, the sections were dehydrated and set up in a slide in a standard medium. Negative controls were carried out without the primary antibody. The vascular endothelium, in which the expression of both annexins had previously been shown, was used as a positive control.

Since staining showed a homogeneous distribution, a semiquantitative scoring system based on staining intensity was applied. Immunostaining was scored blinded to clinical data by two independent observers as negative (0), weak to moderately (1+), and strongly positive (2+) based on staining intensity. Scores ≥ 1 were considered as positive expression.

## 3. Results

### 3.1. Expression of ANXA9 and ANX10 in Normal Epithelia

**Figure 1:**
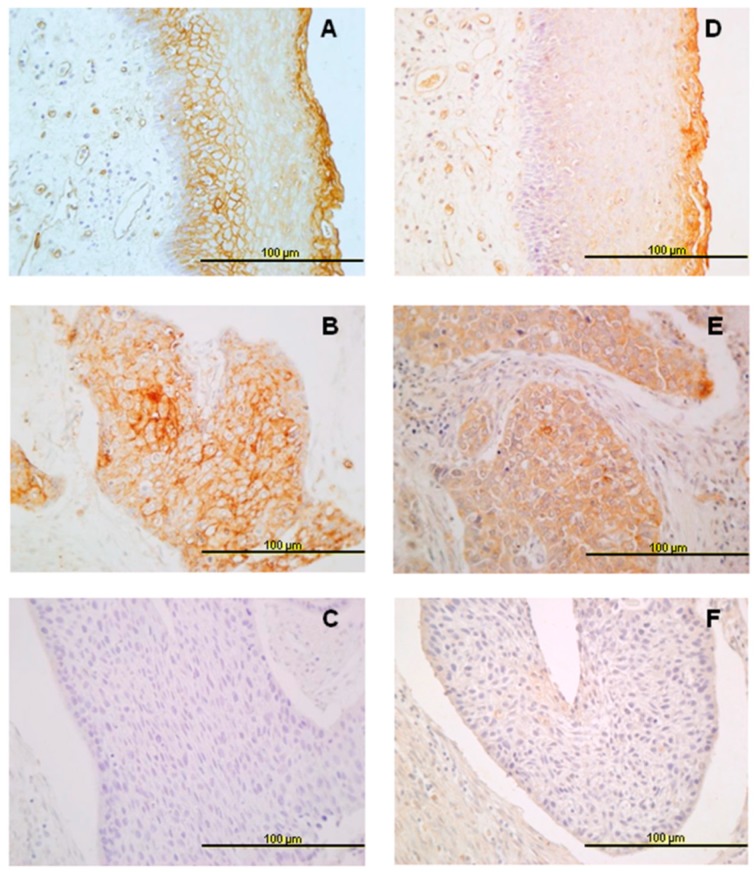
Immunohistochemical analysis of annexins A9 (ANXA9) and A10 (ANXA10) expression in head and neck squamous cell carcinomas (HNSCC) tissue specimens. Representative examples of ANXA9 (**A**) and ANXA10 (**D**) expression in normal epithelium, positive ANXA9 (**B**) and ANXA10 (**E**) expression in carcinomas, and negative ANXA9 (**C**) and ANXA10 (**F**) expression in carcinomas. Original magnification ×40.

Non-keratinized stratified squamous epithelium showed different expression patterns for the two annexins studied. ANXA9 expression was absent in basal and parabasal cells, while expression increased towards the most differentiated layers of the epithelium (Figure 1A). Contrasting this, negative ANXA10 expression was detected in all cell layers of normal epithelium (Figure 1D).

### 3.2. Expression of ANXA9 in HNSCC Tissue Specimens

**Figure 2:**
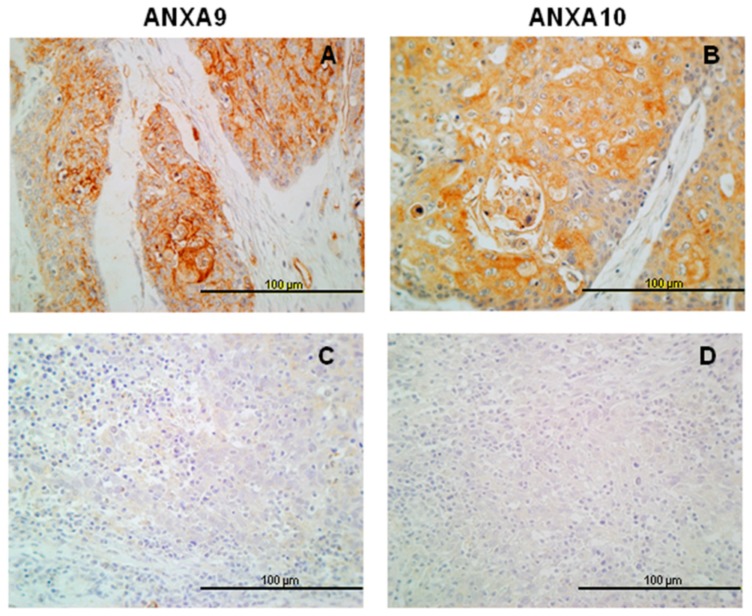
ANXA9 and ANXA10 protein expression in HNSCC specimens according to the degree of differentiation. Representative examples of well-differentiated tumors showing positive expression of ANXA9 (**A**) and ANXA10 (**B**), and poorly differentiated tumors showing negative expression of ANXA9 (**C**) and ANXA10 (**D**) expression in carcinomas. Original magnification ×40.

Immunohistochemical analysis of ANXA9 expression was successfully evaluated in 346 of 372 tumor samples. Two-hundred of them (58%) showed positive ANXA9 expression predominantly with a membranous pattern, although cytoplasmic expression was also observed in some cases (Figure 1B,C). The relationship between the expression of ANXA9 and clinicopathologic characteristics is shown in Table 2. Positive ANXA9 expression was strongly and significantly associated with the degree of differentiation of the tumors (*p* < 0.001). Thus, ANXA9 expression was mainly found in well-differentiated tumors whereas expression was reduced in moderately and poorly differentiated tumors (Figure 2A,C). We also observed differences in ANXA9 expression between the different HNSCC subsites, with ANXA9 expression being significantly higher in oropharyngeal tumors (*p* < 0.001).

No associations were found between ANXA9 expression and T and N classifications or tumor recurrence (*p* = 0.91). In addition, ANXA9 expression was not associated with disease-specific survival (log rank *p* = 0.497) nor overall survival (log rank *p* = 0.406) (data not shown).

### 3.3. Expression of ANXA10 in HNSCC Specimens

Immunohistochemical ANXA10 expression was successfully evaluated in 340 of 372 tumor samples. Positive ANXA10 expression was observed in a total of 219 (64%) cases, mainly detected in the cytoplasm of cancer cells (Figure 1E,F). Furthermore, ANXA9 and ANXA10 expression were significantly correlated (Spearman correlation coefficient 0.459, *p* < 0.001).

Similar to ANXA9, ANXA10 expression was significantly higher in oropharyngeal tumors (*p* = 0.019). Also, ANXA10 expression was significantly associated with the degree of differentiation of the tumors (decreased expression with dedifferentiation, *p* < 0.001, Figure 2B,D). No associations were observed with T and N classifications, disease stage, or tumor recurrence (Table 2). In addition, ANXA10 expression was not associated with either disease-specific (log rank *p* = 0.077) or overall survival (log rank *p* = 0.167).

### 3.4. In Silico Analysis of ANXA9 and ANXA10 mRNA Expression Using The Cancer Genome Atlas (TCGA) HNSCC Data

**Figure 3:**
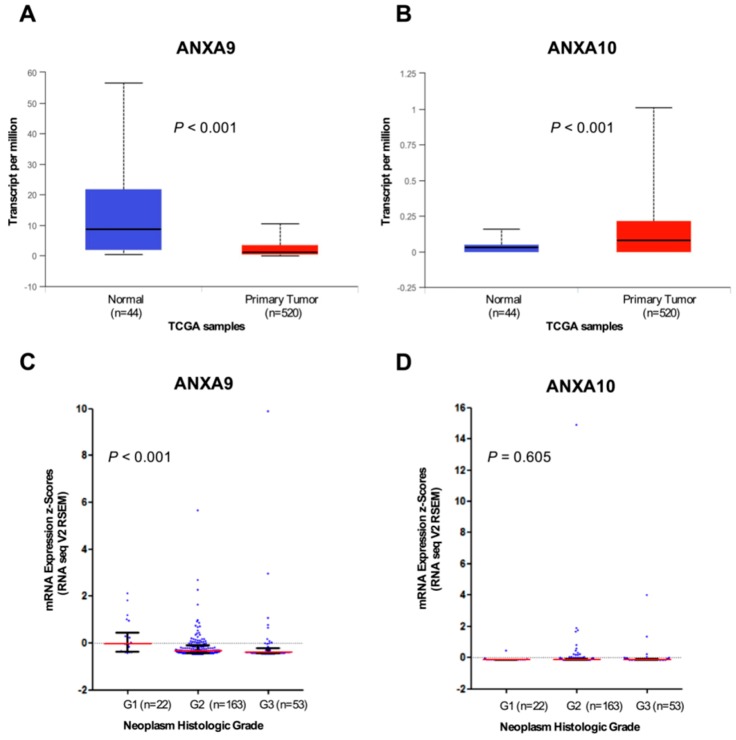
Analysis of ANXA9 and ANXA10 mRNA expression using RNAseq data from the TCGA HNSCC cohorts. Box plots comparing the mRNA expression levels of ANXA9 (**A**) and ANXA10 (**B**) in primary tumors (in red) versus normal tissue (in blue) using UALCAN online resources (http://ualcan.path.uab.edu/). The median, quartiles and range of values are shown. ANXA9 (**C**) and ANXA10 (**D**) expression (RNA seq V2 RSEM, z-score threshold ±2) was analyzed in relation to the tumor grade, categorized as well-differentiated (G1), moderately differentiated (G2) and poorly differentiated (G3) using the TCGA HPV-negative HNSCC cohort (*n* = 243). Horizontal lines (in red) represent the median values, with interquartile range. Sigma (two-tailed) *p*-values.

In order to extend and confirm our results, we also performed analysis of the transcriptome data from the TCGA HNSCC cohort accessed via the original publication [22], or using the platform cBioPortal for Cancer Genomics (http://cbioportal.org/) [23] and the UALCAN web tools (http://ualcan.path.uab.edu/) [24]. Thus, ANXA9 mRNA levels were found to be significantly decreased in primary tumors compared to normal tissue samples (*p* < 0.001; Figure 3A), whilst ANXA10 mRNA levels increased in tumors versus normal tissue (*p* < 0.001; Figure 3B). These results are in good agreement with our observations at the protein level. In addition, possible correlations between ANXA9 and ANXA10 mRNA expression and the tumor grade were assessed using a homogeneous cohort of 243 HPV-negative HNSCC patients. We found that ANXA9 mRNA levels inversely and significantly correlated with the degree of histological differentiation (Spearman correlation coefficient −0.244, *p* < 0.001; Figure 3C). Consistent with our IHC protein data, ANXA9 mRNA levels were higher in well-differentiated tumors than in moderately and poorly differentiated tumors. However, ANXA10 mRNA levels did not significantly correlate with the tumor grade (*p* = 0.605; Figure 3D).

## 4. Discussion

Annexins are commonly altered in cancers [9,25]. ANXA9 is a unique member of the annexin family whose intracellular activity does not appear to be regulated by calcium [10,26]. Its closest evolutionary relatives are ANXA1 and ANXA2 [1,4] and members of this clade are thought to function in the organization and regulation of membrane/cytoskeleton linkages [4,27]. As both ANXA1 and ANXA2 have been found down-regulated in head and neck squamous cell carcinoma [28,29,30], it was of special interest to determine whether ANXA9 showed a similar pattern of expression as this might relate to common features in the evolution, structure and function of these clade members.

We observed a weak membranous ANXA9 expression in the most differentiated cells in normal epithelium. In tumor cells, the expression is mainly membranous, similar to that observed for ANXA2 [28] and the expression of ANXA9 is mainly associated with the degree of differentiation of the tumor, with higher expression in well differentiated cases. This is consistent with elevated ANXA9 observed in differentiating keratinocytes [15]. However, ANXA9 expression was not associated with any other clinical and pathological parameter or with the prognosis in head and neck carcinomas. Analogous findings were obtained by analyzing RNAseq data from the available TCGA HNSCC cohorts. Accordingly, ANXA9 down-regulation was frequently detected in HNSCC at both mRNA and protein levels. Moreover, ANXA9 mRNA expression in tumors was inversely correlated with the histological differentiation grade, thus confirming our IHC protein data. Hence, together these results reflect that transcriptional regulatory mechanisms contribute to the loss of ANXA9 expression in HNSCC, as we previously demonstrated for the functionally and evolutionary-related members ANXA1 and ANXA2 [29,30].

Few studies have analyzed the expression of ANXA9 in cancers. One study showed that *ANXA9* gene expression is associated with bone metastasis in breast cancer [31]. In colorectal cancer, patients with high *ANXA9* gene expression also had lower overall survival [32]. ANXA9 protein expression in colorectal cancer was higher than in normal mucosa, and associated with invasion and lymphatic metastasis and, consequently, a worse prognosis [13]. These studies suggest a role for ANXA9 in invasion and metastasis, but this role could not be confirmed in head and neck cancers.

Several studies have identified ANXA10 as a tumor suppressor, diagnostic marker, potential therapeutic target, or prognostic factor in various malignancies, including bladder cancer, hepatocellular carcinoma, acute myeloid leukemia, gastric carcinoma, oral squamous cell carcinoma, pancreatobiliary adenocarcinoma, and urothelial carcinoma [33,34,35,36,37]. Studies have shown that ANXA10 was down-regulated in hepatocellular carcinoma and was associated with a poor prognosis [34,35]. ANXA10 has recently been identified as a marker with high specificity for the serrated histology of colorectal cancer [33,38]. The physiological importance of abundant ANXA10 expression specific to the stomach mucosa and intestinal M-cells is currently unknown.

Only one previous study has analyzed ANXA10 in head and neck cancer; Shimizu et al. [17] showed that ANXA10 is overexpressed frequently in oral squamous cell carcinomas and that this overexpression is associated with tumor size. They suggested that ANXA10 expression may be associated with tumor progression by promoting cell-cycle progression in the G1 phase through activation of the ERK/MAPK signaling pathway, leading to decreased expression of cyclin-dependent kinase inhibitors (CDKIs). While further studies are needed to study the interaction of ANXA10 and the ERK/MAPK signaling pathway, these data suggested that ANXA10 plays an important role in cellular proliferation.

We also observed that ANXA10 was not visibly expressed in normal epithelium, while it was variably expressed in the cytoplasm of cancer cells. Consistent with this, analysis of the transcriptome data from the TCGA HNSCC also demonstrated the up-regulation of ANXA10 mRNA expression in tumors compared to the corresponding normal tissue. In addition, we found that ANXA10 expression, as ANXA9, was lower in poorly differentiated tumors, but it was not related to other clinicopathologic parameters or prognosis. However, we were unable to confirm the correlation of ANXA10 protein expression with the histological grade using RNAseq data. Nevertheless, these apparently contradictory results may reflect the contribution of additional regulatory mechanisms (e.g., translational or post-translational) leading to the frequent up-regulation of ANXA10 protein in over 60% of tumor samples.

## 5. Conclusions

These original results indicate that the expression of annexins A9 and A10 is frequently altered in HNSCC at both mRNA and protein level, suggesting that they could be implicated in the pathogenesis or compensatory mechanisms of these cancers. Additional studies are ongoing to establish the pathogenic roles of these proteins in the progression of squamous cell carcinomas of the head and neck and especially, to determine whether their altered expression is a cause or consequence of the cancerous state. The association of ANXA9 with pathogenic prognosis in colorectal cancer [13] contrasts with a proposed tumor suppressor role for ANXA10 in gastric cancer [36]. The unique, calcium-independent actions of these two annexins may also contribute to a better understanding of their underlying mechanisms. Since these particular annexins are poorly expressed in general but exhibit highly tissue-specific expression, it will undoubtedly be important to explore the role of epigenetic regulatory changes responsible for their selective expression in normal versus cancer tissues.

## Figures and Tables

**Table 1:** Clinicopathologic characteristics of the tumors studied.

Characteristic	No. Cases (%)
Age, mean (range)	58.6 (30–86 years)
Location	
Oropharynx	241 (65)
Hypopharynx	64 (17)
Larynx	67 (18)
pathologic T classification	
T1	38 (10)
T2	77 (21)
T3	125 (34)
T4	132 (35)
pathologic N classification	
N0	103 (28)
N1	46 (12)
N2	183 (49)
N3	40 (11)
Stage	
I	20 (5)
II	24 (6)
III	64 (17)
IV	264 (71)
Degree of differentiation	
Well-differentiated	147 (39)
Moderately-differentiated	148 (40)
Poorly-differentiated	77 (21)
Total	372

**Table 2:** Relationship between ANXA9 and ANXA10 expression and clinicopathological parameters.

Characteristic	No. Cases for ANXA9	Positive ANXA9 Expression (%)	*p*	No. Cases for ANXA10	Positive ANXA10 Expression (%)	*p*
Location						
Oropharynx	234	166 (71)		231	160 (69)	
Hypopharynx	58	17 (29)	0.000 #	55	28 (51)	0.019 #
Larynx	54	17 (31)		54	31 (57)	
pT Classification						
T1-T2	100	52 (52)		95	58 (61)	
T3	120	73 (61)	0.377 #	119	75 (63)	0.591 #
T4	126	73 (59)		123	83 (67)	
pN Classification						
N0	87	48 (55)	0.616 †	87	53 (61)	0.439 †
N1-3	259	152 (59)		253	166 (66)	
Stage						
I-II	33	14 (42)		32	19 (59)	
III	61	39 (64)	0.124 #	60	39 (65)	0.822 #
IV	252	147 (58)		248	161 (65)	
Degree of differentiation						
Well-differentiated	136	98 (72)		134	103 (77)	
Moderately-differentiated	137	73 (53)	0.000 #	137	85 (62)	0.000 #
Poorly-differentiated	73	29 (40)		69	31 (45)	
Recurrence						
No	132	77 (58)	0.91 †	132	80 (61)	0.248 †
Yes	214	123 (57)		208	139 (67)	
Total	346	200 (58)		340	219 (64)	

# Chi-square and † Fisher’s exact tests.
